# Preface

**Published:** 1884

**Authors:** 


### PREFACE.

COUGH, with its attendant aches and pains, debility and emaciation, is one of the commonest complaints with which the physician has to deal. No excuse is therefore necessary for the appearance of a volume whose sole aim is to assist the busy physician in treating a complaint so frequently met with, and often in such intractable forms. The numerous cough symptoms, before scattered through many volumes, are now for the first time brought together and properly arranged, so that the physician may readily find symptoms which before would have taken hours to discover.

The object, then, of this repertory is to include under one head, and so to render more available, all the valuable and reliable symptoms connected with cough and expectoration found hitherto scattered through many volumes. To this end a careful review has been made of the works of the most reliable authorities of the homœopathic school. A list of these is added.

To properly account for the reliability of all the symptoms that have been collected, they have been numbered, so one can easily refer to the authority responsible for them.

It will be noticed in large blocks of remedies that there are often many different numbers used. The superior figure (over any remedy) tells from what authority that remedy was taken; the number at the end of a block signifies that all remedies, excepting those otherwise numbered (as by a superior figure), are from the author to whom the figure refers. *All* unnumbered symptoms, as well as those marked 1, are from Allen's *Encyclopœdia.*

To secure ready access to the symptoms, they have been arranged alphabetically, according (1) to the anatomical part affected, (2) under the name of the cough, (3) with reference to the time in which it occurs or is aggravated, and (4) under the exciting cause.

Some symptoms are so complex that it is a very difficult matter to decide as to how they should be placed; these have generally been arranged with reference to their most peculiar feature.

A cough caused by a tickling in larynx, will be found under larynx, not under tickling; one causing soreness in stomach, or head, will be found under stomach or head, as the case may be, not under soreness.

In making a prescription for any trouble of the respiratory organs, the concomitant symptoms, especially the mental, are of great importance. Yet we have been compelled to omit all concomitants that are not found in our materia medica under chest, larynx, etc., or which do not frequently occur as a concomitant of cough. For, as any symptom of the materia medica *may* be a concomitant of cough, our task would have extended into many volumes had we included all of them. This repertory is intended to be merely an *index* to the materia medica. Before prescribing, the drug should be studied there.

After the repertory proper had been completed, it was again thoroughly revised and compared with the originals. The result was not only the finding of some misprints, errors, etc., which are noted under the errata, but also the discovery of errors, contradictions, etc., in the original authorities themselves, together with the accumulation of new and important symptoms, not before met with, which were considered too valuable to be neglected, and hence have been included in a supplement.

It is to be hoped all errors of omission or commission, and many such must needs be in a work of this nature, will be promptly reported, so all may be benefited. It is only by such revising and correcting that our therapeutics can be made perfect.

The editors desire to thank all those who have kindly assisted them in their work, and would particularly acknowledge their indebtedness to Drs. Adolph. Lippe and E. W. Berridge for many valuable suggestions.

E. J. L.

2109 Chestnut Street,

Philadelphia, *January 1st, 1884.*

